# Estimating Endmember Backscattering Coefficients Within the Mixed Pixels Based on the Microwave Backscattering Contribution Decomposition Model

**DOI:** 10.3390/s25247587

**Published:** 2025-12-14

**Authors:** Yubin Song, Zhitong Zhang, Hongwei Zheng, Xiaojie Hou, Jiaqiang Lei, Xin Gao, Olaf Hellwich

**Affiliations:** 1 XinJiang Huier Agriculture Group Co., Ltd., Changji 831100, China; houxiaojie@hr.xjher.com; 2 School of Remote Sensing and Information Engineering, North China Institute of Aerospace Engineering, Langfang 065000, China; 3 State Key Laboratory of Desert and Oasis Ecology, Xinjiang Institute of Ecology and Geography, Chinese Academy of Sciences, Urumqi 830011, China; hzheng@ms.xjb.ac.cn (H.Z.);; 4 Research Center for Ecology and Environment of Central Asia, Chinese Academy of Sciences, Urumqi 830011, China; 5 University of Chinese Academy of Sciences, Beijing 100049, China; 6 Computer Vision and Remote Sensing, Technical University Berlin, 10623 Berlin, Germany; olaf.hellwich@tu-berlin.de

**Keywords:** endmembers, hyperspectral unmixing, microwave backscatter contribution decomposition model, radar backscattering coefficient

## Abstract

**Highlights:**

**What are the main findings?**

- A decomposition theory for endmember backscattering contributions is developed.
- A novel estimation scheme for endmember backscattering coefficients is proposed.

**What are the implications of the main findings?**

- Offer a physical basis for unmixing radar signals within mixed pixels.
- Help to construct accurate backscattering-based parameter estimation models.

**Abstract:**

The complexity of land types and the limited spatial resolution of Synthetic Aperture Radar (SAR) imagery have led to widespread mixed-pixel contamination in radar backscatter images. The radar backscatter echo signals from a mixed pixel are often a combination of backscattering contributions from multiple endmembers. The signal mixture of endmembers within mixed pixels hinders the establishment of accurate relationships between pure endmembers’ parameters and the corresponding backscatter coefficient, thereby significantly reducing the accuracy of surface parameter inversion. However, few studies have focused on decomposing and estimating the pure backscatter signals within mixed pixels. This paper proposes a novel approach based on hyperspectral unmixing techniques and the microwave backscatter contribution decomposition (MBCD) model to estimate the pure backscatter coefficients of all Endmembers within mixed pixels. Experimental results demonstrate that the model performance varied significantly with endmember abundance. Specifically, high accuracy was achieved in estimating soil backscattering coefficients when vegetation coverage was below 25% ($R^{2}\approx 0.88$, with 98% of pixels showing relative errors within 0–20%); however, this accuracy declined as vegetation coverage increased. For grass endmembers, the model maintained high estimation precision across the entire grassland area (vegetation coverage 0.2–0.8), yielding an of 0.80 with 83% of pixels falling within the 0–20% relative error range. In addition, the model performance is influenced by the number of endmembers.

## 1. Introduction

Since the 1960s, SAR has been utilized for Earth observation. Today, remote sensing radar has become a standard tool, with its applications steadily expanding into various fields. These include tectonic and lithological mapping in geology [1]. Soil moisture estimation, watershed delineation, flood mapping, surface water, and snow monitoring in hydrology [2,3,4,5]. Crop mapping, growth and harvest monitoring, stress detection, and water resource assessment in agriculture [6,7]. Deforestation monitoring, wildfire damage assessment, and vegetation density estimation in forestry [8,9,10,11]. Topographic mapping, land use classification, landmark change detection, and urban development monitoring in cartography [12,13,14]. And sea ice monitoring, iceberg detection and tracking, glacier and ice sheet mapping, and glacier dynamics observation in polar research [15,16,17]. Unfortunately, most of the aforementioned applications based on radar backscatter coefficients suffer from mixed-pixel signals. The signal mixture makes it difficult to establish a reliable relationship between the surface target’s pure backscatter response and its associated biophysical parameters, thereby limiting the accuracy of parameter retrieval.

There is limited research on the decomposition and estimation of backscattered power from endmembers within mixed pixels. Among the limited related studies, polarimetric decomposition of the backscatter matrix is one of the few approaches that address this issue. This technique decomposes the backscattering matrix into surface, volume, and double-bounce scattering components, thereby providing insights into the endmembers contributing to the observed backscattered energy.

Methods for polarimetric scattering decomposition can be broadly categorized into three types. The first type, proposed by Freeman and Durden under the assumption of reflection symmetry, decomposes the polarimetric SAR backscatter matrix into a linear combination of volume, surface, and double-bounce scattering. Using fully polarimetric SAR observations, the scattering powers of these three components are estimated, which, in turn, characterize the Earth’s surface target and its parameters [18]. Yamaguchi introduced a helix-scattering term into the model, expanding the scattering mechanisms from three to four categories, thereby enhancing the model’s ability to describe complex, high-scattering targets [19]. Shubham replaced Volume Power with Depolarized Volume Power (DVP) and Anisotropic Volume Power (AVP) to improve polarization’s sensitivity to vegetation scattering. Although this approach can enhance the accuracy of Leaf Area Index (LAI) inversion to some extent, it cannot fundamentally distinguish the volume scattering contributions of different endmembers within mixed pixels [20]. Ainsworth proposes a new approach to model-based decomposition by employing an L1-regularized optimization procedure that automatically selects a set of optimal polarimetric scattering mechanisms and guarantees nonnegative powers for the selected mechanisms [21]. Progress in this category of methods has been modest, with current research primarily focused on refining internal-scattering models to improve the accuracy of scattering-power estimates for individual scattering mechanisms. The second category is represented by the method proposed by Cloude and Pottier, which is entirely based on eigenvector decomposition. This approach decomposes the backscattering matrix into entropy, anisotropy, and alpha angle to characterize different scattering mechanisms. Through this decomposition, the dominant scattering mechanism and the associated scattering power can be inferred from the principal eigenvalue and its corresponding eigenvector [22]. Mott made approximations to interpret the eigenvector-based decomposition results regarding known scattering mechanisms [23].

These traditional backscattering power decomposition methods, including eigenvalue and polarimetric decomposition, successfully resolve the backscattering power of mixed pixels into canonical scattering mechanisms (surface, volume, and double-bounce). However, different endmembers within a mixed pixel often exhibit overlapping scattering behaviors; for example, trees and grass both contribute to the volume scattering component. Furthermore, a single endmember in a mixed pixel can contain multiple scattering mechanisms, as seen in trees, including both canopy-surface and internal-volume scattering. Consequently, since the backscattering of most natural land types is a composite of multiple mechanisms, traditional mechanism-based decomposition methods are insufficient for isolating the energy contributions of distinct endmembers.

By combining hyperspectral unmixing with MBCD models, the problem of endmember backscattering energy decomposition, which traditional methods cannot address, is expected to be solved. In our study on desertification in arid and semi-arid regions, we proposed the MBCD model within mixed pixels containing only two endmembers: soil and vegetation. Additionally, we developed a buffer-based approach for estimating the backscattering coefficients of individual endmembers [24]. The buffer-based approach for estimating endmember backscattering coefficients requires endmember abundances within mixed pixels as prior information. In our previous work, vegetation abundance was derived using the vegetation coverage method, and soil abundance was indirectly inferred [24]. However, this approach becomes inadequate when mixed pixels contain three or more endmembers, as it cannot retrieve the abundances of all endmembers. Therefore, this study proposes integrating hyperspectral unmixing techniques to provide abundant information for estimating endmember backscattering coefficients. Combining hyperspectral unmixing with the buffer-based estimation approach enables estimation of the backscattering coefficients of individual endmembers within arbitrarily complex mixed pixels.

Current methods for extracting pure endmembers from hyperspectral imagery can be broadly classified into four categories: spectral library-based, visual interpretation, geometrical approaches, and deep learning-based techniques [25,26,27,28,29,30,31,32,33].

Visual interpretation of optical imagery relies on prior knowledge of the ground surface’s spectral characteristics and textures, enabling users to select representative pixels as pure endmembers manually. Among geometrical approaches, the N-FINDR algorithm and its variants are the most representative [34]. These algorithms assume that end members form a simplex of maximum volume in the feature space. By identifying the set of pixels that define this maximum-volume simplex, the algorithms aim to extract pure endmembers from the hyperspectral data. Another classical geometrical method is the Pixel Purity Index (PPI) [35]. This method is based on projection analysis, where image pixels are projected onto many random vectors. The frequency with which a pixel appears at the extremes of these projections is recorded, with pixels appearing most frequently at extreme positions considered more likely to represent pure endmembers. In recent years, with the advancement of deep learning techniques and the increasing availability of labeled samples, deep learning has been gradually applied to endmember extraction and has demonstrated relatively promising performance [36,37,38,39,40].

Commonly used spectral unmixing methods can be broadly categorized into two main types: Physics-Based and Data-Driven spectral unmixing [41]. Data-driven hyperspectral unmixing methods aim to directly learn the mapping between endmember spectra and abundances from observed data without relying on predefined physical models or spectral assumptions [42,43,44]. Physics-based spectral unmixing methods interpret the composition and proportions of mixed pixels by constructing mathematical models grounded in physical laws governing the interactions between light and matter [45,46,47,48]. Data-driven hyperspectral unmixing methods rely heavily on large volumes of high-quality training data. They may exhibit limited generalization capability when faced with high-dimensional features, noise, or significant endmember variability. Physically based nonlinear spectral unmixing methods can more accurately model complex interactions among surface materials; however, their high model complexity and difficulty in parameter estimation often lead to greater uncertainty in derived abundance estimates. In contrast, linear models offer a simpler structure and clearer physical interpretability, making them suitable for most practical applications and thus widely adopted.

To address the challenge of mixed pixel effects in SAR backscattering analysis, this study proposes a general technical framework for estimating endmember-level backscattering coefficients within mixed pixels by integrating linear hyperspectral unmixing with the MBCD model, and the focus of this paper, including:

1. Developing the MBCD model, which was initially limited to decomposing backscattering contributions in mixed pixels containing only vegetation and soil, to a generalized model capable of handling mixed pixels with arbitrary types and numbers of endmembers.
2. Proposing a general technical framework to estimate the endmember backscattering coefficients for any mixed pixels.
3. Assessing the performance of the proposed general technical framework for estimating endmember-level backscattering coefficients within mixed pixels.

## 2. Data and Methods

### 2.1. Test Area

**Figure 1:**
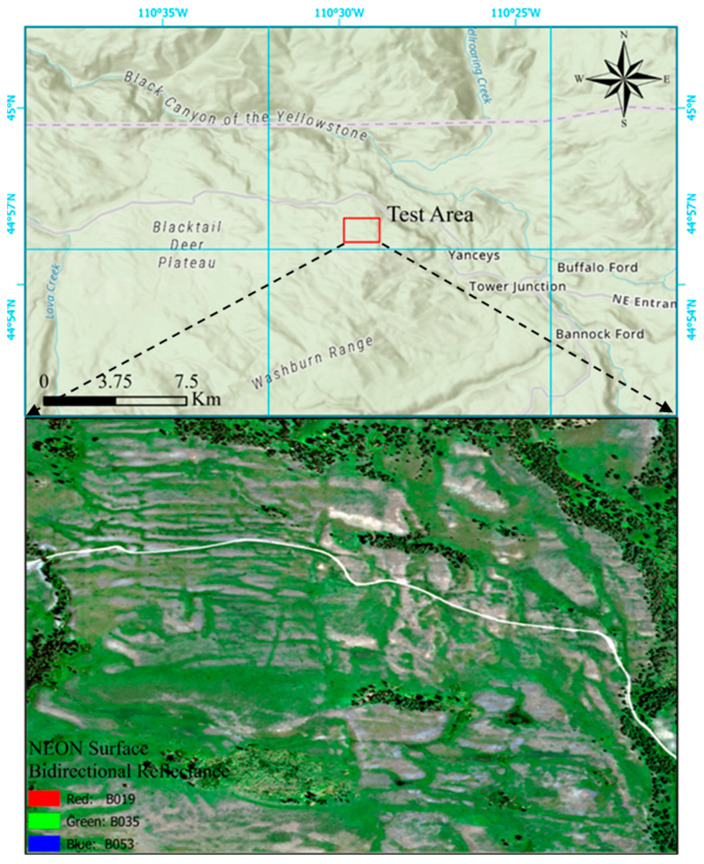
Test Area and the corresponding NEON surface bidirectional reflectance data.

The selection of the test area primarily depends on the availability of hyperspectral data. Although numerous open-access hyperspectral datasets are currently available, most of them are designed for tasks such as spectral unmixing or land cover classification and often lack corresponding spatial location information. Given the completeness of geolocation, the complexity of land cover types, and the computational demands (as hyperspectral datasets typically contain hundreds of spectral bands), we selected a region within Yellowstone National Park as the test site. This area covers approximately 1.67 square kilometers, as shown in Figure 1.

YELL represents a wildland area within the National Ecological Observatory Network (NEON) Northern Rockies Domain. The terrain consists of rolling hills spanning 1840–2245 m (6036–7360 ft) in elevation at the site. The site is a mosaic of pine-dominated forest mixed with open swaths of sage, grass, and small wetlands. Soils at the YELL NEON site fall into the Molli soil category. Most soils found in the area are within loamy-skeletal particle-size families. The most common soil type found in the NEON sample plots was Hobacker gravelly loam [49].

### 2.2. Datasets

#### 2.2.1. Hyperspectral Data

In this study, we used hyperspectral imagery from NEON, specifically the Surface Bidirectional Reflectance data product acquired through the NEON Airborne Observation Platform (AOP). This dataset is derived from visible-to-shortwave infrared (VSWIR) hyperspectral imagery spanning approximately 380 nm to 2510 nm. It consists of 426 spectral bands with a resolution of approximately 5 nm, and the reflectance values are scaled by a factor of 10,000. Wavelength regions corresponding to strong water vapor absorption, namely 1340–1445 nm and 1790–1955 nm, are masked out and assigned a value of −100 due to the lack of valid reflectance information. Additionally, the dataset includes quality-assurance (QA) raster bands to assist in filtering unreliable observations. The dataset underwent a series of preprocessing steps to ensure geometric and radiometric accuracy, including orthorectification and corrections for atmospheric effects, topographic effects, and the Bidirectional Reflectance Distribution Function (BRDF). The final reflectance mosaic was constructed using nadir-most pixels from flight lines with minimal cloud cover. Hyperspectral data of the test area, acquired on 4 July 2023, were obtained from the Google Earth Engine (GEE) platform and exported to the local disk for subsequent processing.

#### 2.2.2. Microwave Backscatter Data

Microwave backscatter data were obtained from the Sentinel-1A mission, which carries a C-band SAR instrument operating at 5.405 GHz. The Ground Range Detected (GRD) products were preprocessed using the Sentinel-1 Toolbox to generate terrain-corrected and radiometrically calibrated backscatter data. The preprocessing pipeline included thermal noise removal, radiometric calibration, and terrain correction based on the Shuttle Radar Topography Mission (SRTM) 30 m Digital Elevation Model (DEM), or ASTER DEM in high-latitude regions where SRTM is unavailable. The resulting backscatter coefficients were converted to decibel (dB) values using a logarithmic scale transformation. This study used GRD products acquired in the ascending orbit mode with single-polarization VV (vertical transmit and receive). The test area data, acquired on 4 July 2023, was retrieved from the GEE platform.

### 2.3. Methods

#### 2.3.1. A General Technical Framework for Estimating the Backscatter Coefficients of Endmembers Within Mixed Pixels

**Figure 2:**
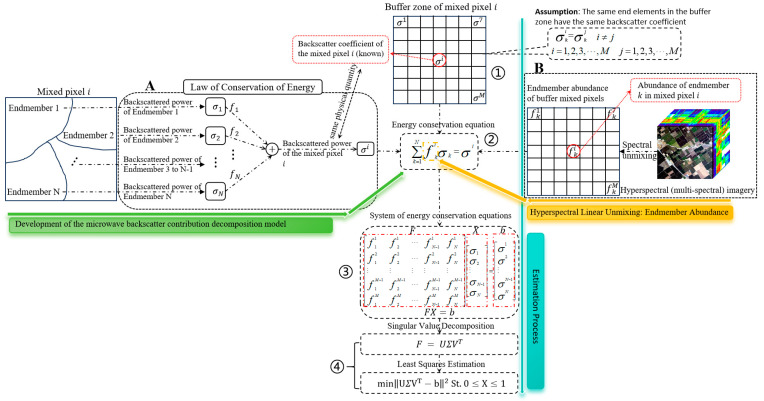
The technical framework for estimating the backscatter coefficients of endmembers within mixed pixels. A detailed account of the methodology is presented in Section 2.3.1.

Figure 2 illustrates the overall technical framework for estimating endmember backscatter coefficients in mixed pixels proposed in this study. As shown in Part A of Figure 2, building on our previous work, we first extend the two-endmember MBCD model to a multiple-endmember MBCD model [24]. This extension is derived through rigorous theoretical analysis, resulting in a backscatter energy conservation Equation applicable to arbitrary mixed pixels. This equation provides the theoretical foundation for estimating endmember backscatter coefficients.

Secondly, we continued to employ the “buffer-based estimation approach” proposed in our previous work, assuming that end members of the same class within a buffer share identical backscatter coefficients; this approach remains applicable to multiple members (Step ①, as shown in Figure 2). Based on the multi-endmember MBCD model, an energy conservation equation can be established for each mixed pixel within the buffer (Step ②, as shown in Figure 2). Since the abundance of the same end member is usually different across different mixed pixels in the buffer, it becomes possible to construct a system of energy conservation Equations encompassing all mixed pixels within the buffer zone (Step ③, as was shown in Figure 2).

Finally, singular value decomposition (SVD) is applied to the abundance matrix to obtain a least-squares estimate of the endmember backscatter coefficient vector for mixed pixels. When two mixed pixels in the buffer exhibit highly similar endmember types and abundances, the abundance matrix may become singular, resulting in low estimation accuracy. SVD helps mitigate this issue by improving the numerical stability and computational efficiency of the solution. Furthermore, we noted that the normalized backscattering coefficients range from 0 to 1; therefore, the solutions to the system of energy balance equations must also fall within this range (Step ④, as was shown in Figure 2).

#### 2.3.2. Endmember Abundance Estimation via Linear Spectral Unmixing

##### Linear Spectral Mixture Model (LSMM)

**Figure 3:**
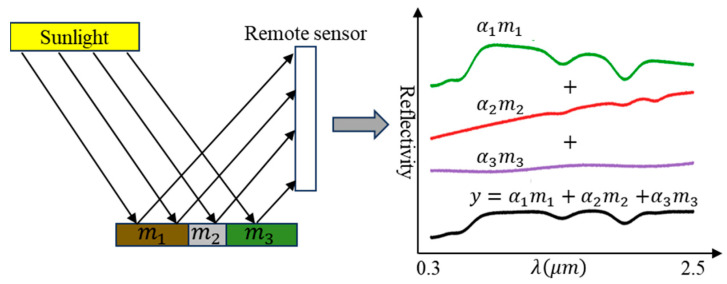
The diagram of the physical principle of the Linear Spectral Mixture Model (adapted from [41]).

Figure 3 illustrates the physical principle of the Linear Spectral Mixture Model (LSMM). Assuming negligible multiple scattering interactions, the observed spectrum y is expressed as the weighted sum of individual endmember spectra ($m_{1},m_{2},m_{3}$) present within the pixel. The weights correspond to the fractional area occupied by each endmember ($\alpha_{1},\alpha_{2},\alpha_{3}$), subject to non-negativity and sum-to-one constraints.

In contrast to non-linear spectral mixture models and deep learning-based methods, the LSMM offers distinct advantages, including simplicity, high computational efficiency, and explicit physical interpretability. Particularly for hyperspectral imagery with a spatial resolution lower than the meter level, the LSMM more accurately characterizes actual spectral mixing phenomena [50]. Therefore, this study uses the LSMM to estimate endmember abundances.

##### Acquisition of Pure Endmembers and Corresponding Spectral Features

Given the relatively small spatial extent of the study area, the low spectral–spatial variability among similar endmembers, and the limited number of pure pixels, traditional methods such as PPI and N-FINDR may fail to accurately identify pure pixels, as they are sensitive to threshold settings. To address this issue, we adopted a method based on visual interpretation, combined with the Normalized Difference Vegetation Index (NDVI), to identify pure pixels and extract their representative spectral signatures.

The specific procedure is as follows:

(1) NDVI values were extracted for the study area and subjected to density segmentation. (2) Pure Tree and grass pixels were identified by iteratively adjusting the NDVI threshold range. Theoretically, a larger vegetative cover corresponds to higher chlorophyll content and, thus, higher NDVI values. Candidate regions for Tree and grass were first visually identified using high-resolution imagery, and a lower NDVI threshold was then applied to isolate spectrally pure Tree and grass pixels. (3) Pure soil endmembers were determined using the upper NDVI threshold. In theory, chlorophyll content decreases as soil fraction increases, leading to lower NDVI values. Visually identifiable pure soil regions were used as a reference, and an appropriate upper NDVI limit was applied to extract pure soil pixels. (4) Roads within the study area are primarily composed of cement and exhibit distinct spectral characteristics, allowing pure road pixels to be visually identified from the RGB imagery.

##### Fully Constrained Least Squares (FCLS) Spectral Unmixing Model

The FCLS algorithm was initially proposed by Heinz in 2001, and this method assumes that the observed spectrum of a given pixel can be expressed as a linear combination of endmember spectra [45]:

(1) r = Mα + n

Here, r is an N×1 vector representing the reflectance of a mixed pixel across N spectral bands; **M** is an N × p matrix corresponding to the reflectance spectra of p endmembers; **α** is a p × 1 vector denoting the abundance of the endmembers within the mixed pixel; n is an N × 1 vector representing the noise. Therefore, the estimation of endmember abundances in a mixed pixel can be expressed as:

(2) $$\min\left\{{\left(r-M\alpha \right)}^{T}\left(r-M\alpha \right)\right\}\;\text{Subject to}:(1)\;\alpha_{i}\;\geq \;0\;\left(I\;=\;1,\;\cdots ,\;p\right)\;(2)\;\sum_{i=1}^{p}\alpha_{i}=\;1$$

#### 2.3.3. Development of the MBCD Model

Assume that the total area of a mixed pixel is S, which contains M types of endmembers. The area occupied by the *i*-th endmember is denoted as $S_{i}$. The backscatter coefficient of this mixed pixel is denoted by σ, while $\sigma_{i}$ represents the backscatter coefficient of the *i*-th endmember to be estimated, where *i* is a positive integer ranging from 1 to M. It is assumed that a total of N mixed pixels within the buffer zone are used to estimate the endmember backscatter coefficients. According to the definition of the backscatter coefficient, the backscatter coefficient of a mixed pixel is the ratio of the backscattered power to the incident power.

(3) $$\sigma =\frac{P_{\mathrm{backscattering}}}{P_{\mathrm{incidence}}}$$

In the above Equation (3), $P_{\mathrm{backscattering}}$ denotes the backscattered power, $P_{\mathrm{incidence}}$ represents the incident power, and *σ* is the backscatter coefficient of this mixed pixel. By performing a straightforward transformation of Equation (3), the backscattered power of this pixel can be expressed as follows:

(4) $$P_{\mathrm{backscattering}}\;={\;P}_{\mathrm{incidence}}\sigma$$

Similarly, the backscattered power of the *i*-th endmember within this mixed pixel can be expressed as follows:

(5) $$P_{i\text{-backscattering}}\;=\;P_{\mathrm{incidence}}\sigma_{i}$$

Therefore, the backscattered energy of this mixed pixel can be derived from two different perspectives, which, according to the law of energy conservation, should be equal:

(6) $$P_{\mathrm{backscattering}}S\;=\;\sum_{i=1}^{M}P_{i\text{-backscattering}}S_{i}$$

In the above Equation (6), the left side represents the total backscattered energy of the mixed pixel, obtained by multiplying the backscatter power by the pixel area. The right side shows the sum of the backscattered energies from all endmembers within the mixed pixel, representing the total backscattered energy. According to the principle of energy conservation, the physical quantities on both sides of Equation (6) are equivalent and must be equal.

By replacing the backscattered power in Equation (6) with the incident power, we obtain the following Equation:

(7) $$P_{\mathrm{incidence}}\sigma S\;=\;P_{\mathrm{incidence}}\sum_{i=1}^{M}\sigma_{i}S_{i}$$

The incident power on both sides of Equation (7) can be canceled out, resulting in a simplified energy conservation Equation for the mixed pixel:

(8) $$\sigma \;=\;\sum_{i=1}^{M}\frac{S_{i}}{S}\sigma_{i}$$

In the above Equation, $S_{i}/S$ represents the ratio of the area occupied by the *i*-th endmember to the total area of the pixel, which corresponds to the abundance of endmember *i*. By denoting $S_{i}/S=f_{i}$, the final energy conservation Equation for the mixed pixel can be expressed as:

(9) $$\sigma \;=\;\sum_{i=1}^{M}f_{i}\sigma_{i}$$

The energy conservation Equation (Equation (9)) enables the decomposition of backscatter contributions from multiple endmembers within a mixed pixel. It will serve as the fundamental basis for estimating endmember backscatter coefficients within mixed pixels.

#### 2.3.4. Sub-Pixel-Level Backscattering Contributions Estimation

The key factors determining endmembers’ backscatter coefficients include their type, structure, and condition. For natural, spatially adjacent, non-manmade targets (such as soil, trees, or grass), the variations in the factors affecting backscatter (such as vegetation composition, structure, and growth conditions) are typically small. Similarly, for artificial targets in a natural environment that are spatially adjacent (such as buildings or concrete roads), the factors influencing backscatter (such as the material composition and structure of buildings and roads) usually exhibit only minor variations. Therefore, it can be assumed that endmembers of the same type within spatially adjacent mixed pixels share the same backscatter coefficient. Based on this assumption, this study proposes a method for estimating endmember backscatter coefficients within mixed pixels.

A buffer zone with a radius of R is established around the mixed pixel P to be estimated (the buffer radius is chosen to ensure that the composition, structure, and condition of the same type of endmembers within the buffer do not exhibit significant spatial variation). It is assumed that there are N mixed pixels within the buffer, with the backscatter coefficient of the j-th mixed pixel denoted as $\sigma^{j}$, where j is a positive integer ranging from 1 to N. Furthermore, each pixel contains M types of endmembers, and the backscatter coefficient of the *i*-th endmember is denoted as $\sigma_{i}$, where i is a positive integer ranging from 1 to M. Based on Equation (6), a system of linear unmixing Equations for estimating the backscatter coefficients of endmembers within the mixed pixel P can be constructed:

(10) $$f_{1}^{1}\sigma_{1}+f_{2}^{1}\sigma_{2}+,\cdots ,+f_{i}^{1}\sigma_{i}+,\cdots ,+f_{M}^{1}\sigma_{M}=\sigma^{1}\;f_{1}^{2}\sigma_{1}+f_{2}^{2}\sigma_{2}+,\cdots ,+f_{i}^{2}\sigma_{i}+,\cdots ,+f_{M}^{2}\sigma_{M}=\sigma^{2}\;\cdots \;f_{1}^{j}\sigma_{1}+f_{2}^{j}\sigma_{2}+,\cdots ,+f_{i}^{j}\sigma_{i}+,\cdots ,+f_{M}^{j}\sigma_{M}=\sigma^{j}\;\cdots \;f_{1}^{N}\sigma_{1}+f_{2}^{N}\sigma_{2}+,\cdots ,+f_{i}^{N}\sigma_{i}+,\cdots ,+f_{M}^{N}\sigma_{M}=\sigma^{N}$$

For any mixed pixel j within the buffer zone of the mixed pixel P, the abundance of endmember i, denoted as $f_{i}^{j}$, can be estimated using the linear spectral unmixing model described in Section 2.3.2. Meanwhile, the backscatter coefficient $\sigma^{j}$ of mixed pixel j can be directly obtained from the original radar imagery. Therefore, the only unknowns in the system of Equation (10) are the backscatter coefficients of the endmembers within the target mixed pixel P, namely σ_1_, σ_2_, …, $\sigma_{M}$. To facilitate the formulation and solution of Equation (10), we introduce the following notations for its variables:

(11) $$F=\left[\begin{array}{c} \begin{array}{ccc} f_{1}^{1} & f_{2}^{1} & \cdots \\ f_{1}^{2} & f_{2}^{2} & \cdots \\ \vdots & \vdots & \vdots \end{array}\;\;\;\;\;\begin{array}{ccc} f_{i}^{1} & \cdots & f_{M}^{1}\\ f_{i}^{2} & \cdots & f_{M}^{2}\\ \vdots & \vdots & \vdots \end{array}\\ \begin{array}{ccc} f_{1}^{j} & f_{2}^{j} & \cdots \\ \vdots & \vdots & \vdots \\ f_{1}^{N} & f_{2}^{N} & \cdots \end{array}\;\;\;\;\;\begin{array}{ccc} f_{i}^{j} & \cdots & f_{M}^{j}\\ \vdots & \vdots & \vdots \\ f_{i}^{N} & \cdots & f_{M}^{N} \end{array} \end{array}\right]\;X=\left[\begin{array}{c} \begin{array}{c} \sigma_{1}\\ \sigma_{2}\\ \vdots \end{array}\\ \sigma_{i}\\ \vdots \\ \sigma_{M} \end{array}\right]\;b=\left[\begin{array}{c} \begin{array}{c} \sigma^{1}\\ \sigma^{2}\\ \vdots \end{array}\\ \sigma^{j}\\ \vdots \\ \sigma^{N} \end{array}\right]$$

The matrix F contains the abundances of all endmembers within the N pixels of the buffer zone and is referred to as the buffer zone’s abundance matrix. The vector b contains the composite backscatter coefficients of these N mixed pixels and is referred to as the composite backscatter vector. The vector X, which consists of the backscatter coefficients of all endmembers within the target mixed pixel P, is the unknown endmember backscatter coefficient vector. Based on these definitions, the system of Equation (11) can be concisely written as:

(12) FX = b

When two or more mixed pixels within the buffer zone exhibit similar endmember abundance distributions, this may lead to linear dependence among row vectors in the abundance matrix F, thereby preventing the accurate computation of a least-squares solution. In such cases, SVD can be applied to the matrix F. The SVD of the abundance matrix F can be expressed as:

(13) $$F\;=\;U\Sigma V^{T}$$

Based on the singular value decomposition of F, and considering the non-negativity of the backscattering coefficients, the least squares estimation in Equation (12) can be reformulated as:

(14) $${min\|U\Sigma V^{T}X-b\|}^{2}\;\mathrm{St}.\;0\leq X\leq 1$$

The non-negative least squares (NNLS) problem defined in Equation (14) was solved using the Lawson–Hanson algorithm [51] to strictly enforce the non-negativity constraints.

#### 2.3.5. Verification Scheme of Backscattering Contributions Estimation

Direct field measurements of endmember backscattering coefficients are often impractical. Therefore, we propose a relatively simple approach to validate the accuracy of the estimated endmember backscattering coefficients. Taking a mixed pixel composed of soil and grass endmembers as an example, we use the backscattering power of pure soil and grass pixel regions within the buffer zone to approximate the true backscattering coefficients of soil and grass endmembers in the target mixed pixel P as illustrated in Figure 2. Assuming that the buffer zone centered on the target pixel contains N mixed pixels for estimating the endmember backscattering coefficients, and that each mixed pixel has an equal area s, the following relationship holds:

(15) $$\sigma_{\text{pure-soil}}^{1}s+\sigma_{\text{pure-soil}}^{2}s+,\cdots ,+\sigma_{\text{pure-soil}}^{i}s+,\cdots ,+\sigma_{\text{pure-soil}}^{N}s=\sigma_{\text{pure-soil}}\mathrm{Ns}$$

(16) $$\sigma_{\text{pure-grass}}^{1}s+\sigma_{\text{pure-grass}}^{2}s+,\cdots ,+\sigma_{\text{pure-grass}}^{i}s+,\cdots ,+\sigma_{\mathrm{grass}}^{N}s=\sigma_{\text{pure-grass}}\mathrm{Ns}$$

Therefore, for mixed pixels composed solely of soil and grass endmembers, the true values of the endmember backscattering coefficients can be approximated as:

(17) $$\sigma_{\text{pure-grass}}\;=\;\frac{1}{N}\sum_{i=1}^{N}\sigma_{\text{pure-grass}}^{i}$$

(18) $$\sigma_{\text{pure-soil}}=\frac{1}{N}\sum_{i=1}^{N}\sigma_{\text{pure-soil}}^{i}$$

The estimation error of the backscattering coefficient is quantified using relative error:

(19) $$\mathrm{RE}\left(\sigma_{\mathrm{soil}}\right)\;=\;\frac{\left|\hat{\sigma_{\mathrm{soil}}}{-\sigma }_{\text{pure-soil}}\right|}{\sigma_{\text{pure-soil}}}\;\times \;100\%$$

(20) $$\mathrm{RE}\left(\sigma_{\mathrm{grass}}\right)=\frac{\left|\hat{\sigma_{grass}}-\sigma_{\text{pure-grass}}\right|}{\sigma_{\mathrm{pure}\text{-}\mathrm{grass}}}\;\times \;100\%$$

Furthermore, the performance of the proposed scheme was comprehensively assessed through scatter plots, the mean, standard deviation, and the coefficient of determination.

## 3. Results

### 3.1. Pure Endmember Extraction

**Figure 4:**
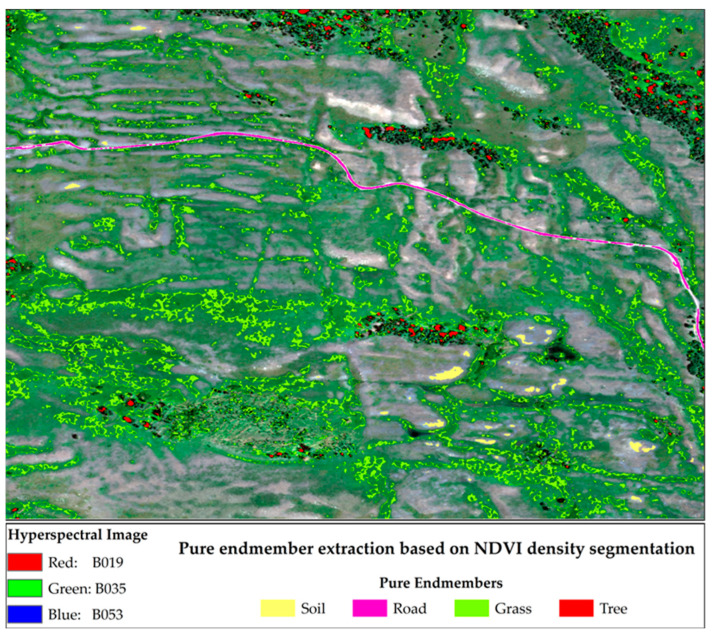
Pure pixel areas containing a single endmember (soil, road, grass, or tree).

Pure endmembers were extracted based on visual interpretation and NDVI values, following the procedure outlined in Section 2.3.1. Specifically, the NDVI range for pure tree endmembers was defined as (0.9600, 1.0000), for pure grass endmembers as (0.8300, 0.8500), and pure soil endmembers as (0.0586, 0.1500). Pure road endmembers were extracted primarily based on visual interpretation, with NDVI values set below 0.1. The results of the extraction for each pure endmember type are presented in Figure 4.

### 3.2. Pure Endmember Spectral Features

**Figure 5:**
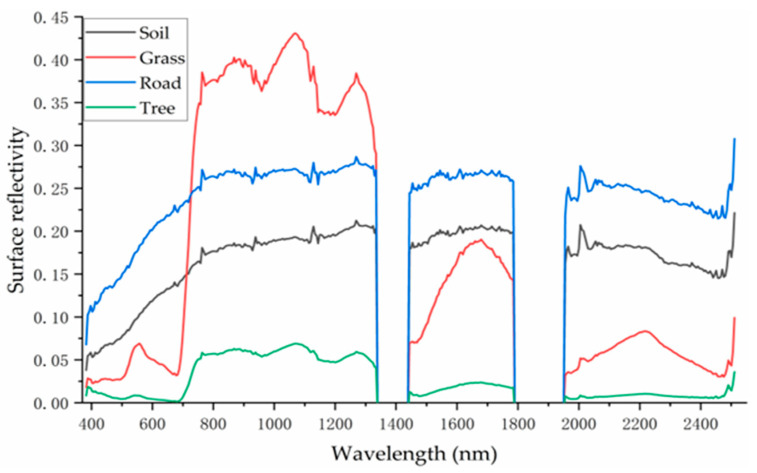
Spectral characteristics curves of pure soil, grass, road and trees.

Based on the four types of pure endmember regions of interest identified in Section 3.1, the spectral signature curves of each class were derived by averaging the reflectance values across all corresponding pure pixels (Figure 5).

As shown in Figure 5, the four classes exhibit distinct spectral characteristics. In the visible wavelength range (400–700 nm), roads show the highest reflectance, followed by soil, grass, and trees. This pattern aligns with the visual impression from the true-color image in Figure 1, where roads appear the brightest, followed by soil, grass, and trees. The relatively low reflectance of trees may be attributed to shadow effects. The consistency between the visual appearance in the visible spectrum and the derived spectral curves supports the reliability and accuracy of the extracted spectral signatures for the four pure endmember types.

### 3.3. Abundance of Pure End Members

**Figure 6:**
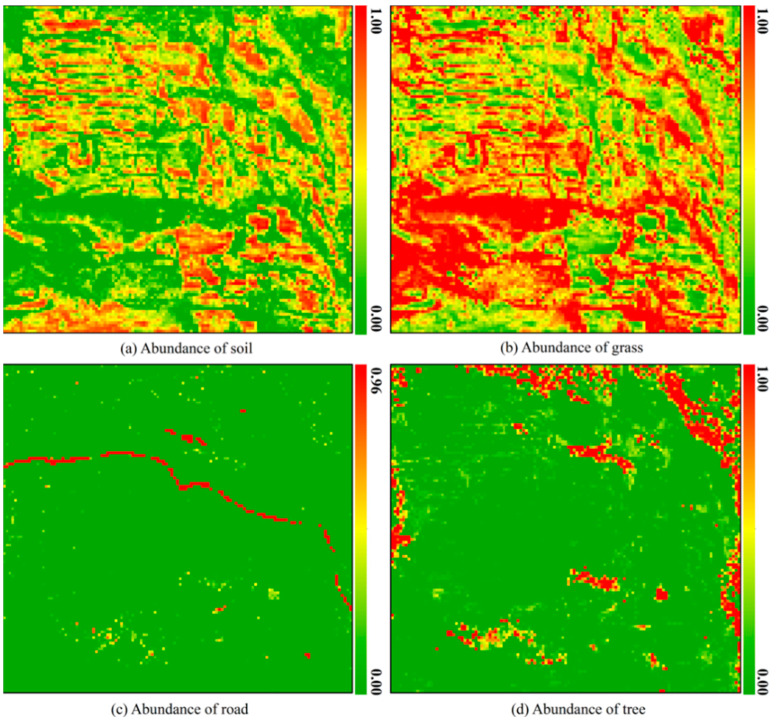
Abundance maps of the four endmembers in the test area.

Based on the spectral signature curves of the four endmember types extracted in Step 3.1, we utilized the FCLS function from the pysptools library (version 0.15.0)—a Python package specialized for hyperspectral data processing—to perform Fully Constrained Least Squares (FCLS) estimation. This algorithm was applied to the hyperspectral imagery of the study area (resampled to a 10 m spatial resolution) to obtain the final endmember abundance estimates, as shown in Figure 6.

The accuracy of abundance estimation for mixed pixels is critical for the subsequent estimation of endmember backscattering coefficients. Therefore, it is essential to evaluate the abundance of unmixing results. However, since ground-truth data on actual endmember abundances are not available for the selected study area, we assess the accuracy of the estimated abundances using both the root-mean-square error (RMSE) from the unmixing process and visual inspection. The RMSE, which is commonly used to evaluate spectral unmixing performance, is defined as follows:

(21) $$\mathrm{RMSE}=\;\sqrt{\frac{1}{N}\sum_{i=1}^{N}{\left(x_{i}-\hat{x_{i}}\right)}^{2}}$$

**Figure 7:**
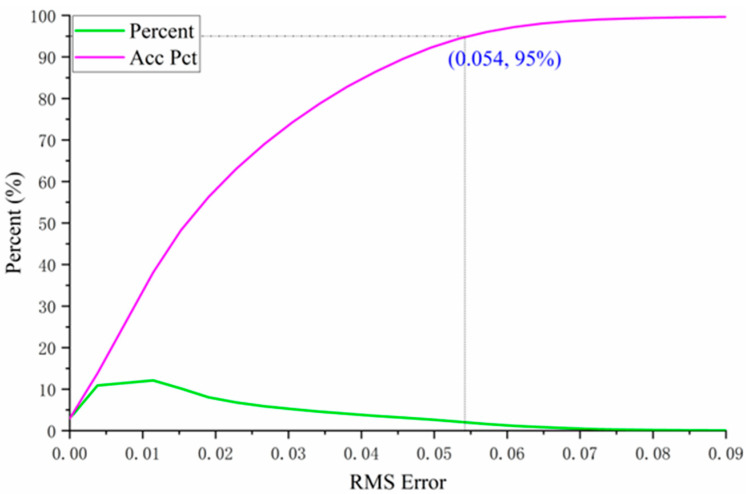
Probability and cumulative probability distribution of RMSE in endmember abundance estimation for the test area. The label (0.054, 95%) indicates that approximately 95% of the samples have a relative error of less than 0.054 in the endmember abundance estimation.

In Equation (21), $x_{i}$ represents the reflectance of the mixed pixel at the *i*th spectral band. At the same time, $\hat{x_{i}}$ denotes the reconstructed reflectance at the same band, obtained by the estimated endmember abundances and the endmember spectral matrix. A smaller RMS indicates better performance in reconstructing the mixed pixel’s spectrum using the estimated abundances, implying lower reconstruction error. Conversely, a larger RMS suggests that the pixel may not fully conform to the linear mixing model, or that the endmember extraction is insufficient or inaccurate. The RMS error distribution of hyperspectral unmixing is illustrated in Figure 7.

### 3.4. Endmember Backscattering Coefficient

**Figure 8:**
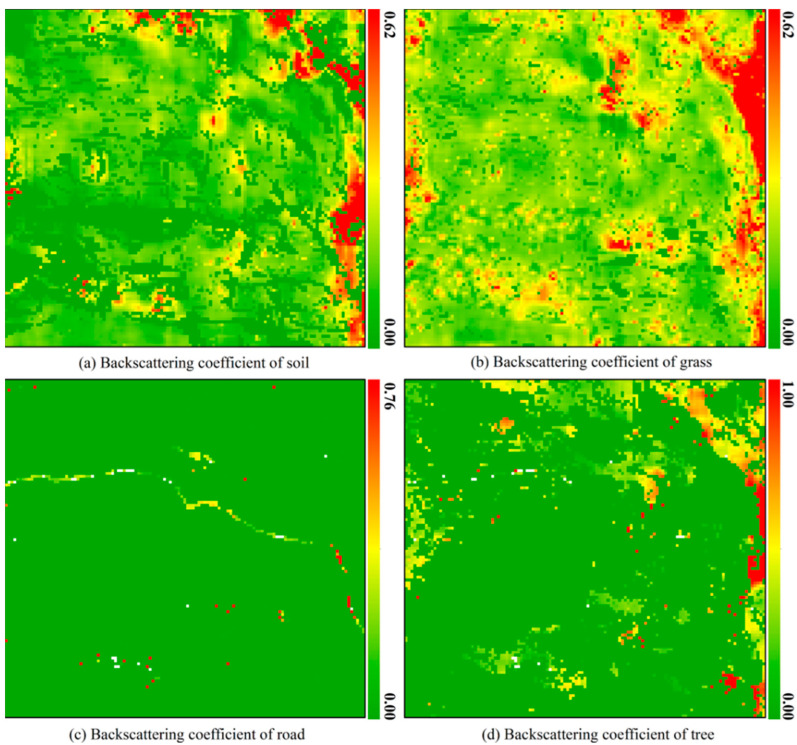
Estimation results of backscattering coefficients of four endmembers in the test area.

For any mixed pixel, when the abundance of an endmember approaches 1, the contributions of the other endmembers become negligible. In such cases, the backscattering energy of the pixel is likely dominated by this single endmember, which can be treated as a pure pixel. The backscattering coefficient of this mixed pixel will be set to the endmember’s value, and the backscattering coefficients of the other endmembers are set to nan. Additionally, in exceptional cases—such as a mixed pixel primarily composed of grass that contains an uncovered strong scatterer or a high-scattering region—the backscattering coefficient of the grass endmember may be significantly overestimated. This issue can be mitigated by applying a spatial median filter to the backscattering coefficient. If estimation is forced under such conditions, the extremely low abundance values of other endmembers may lead to instability and unreliability in the solution of Equation (12). Therefore, during the estimation of endmember backscattering coefficients, a lower bound threshold is imposed on the endmember abundances in Equation (12). When the abundance of any endmember falls below 0.05, the resulting unmixing error can be substantial. Hence, any endmember with an abundance below this threshold within a mixed pixel is considered absent, and its backscattering coefficient is marked as invalid during the estimation process. This study uses a threshold of 0.9 for endmember abundance to identify pure pixels, meaning pixels with endmember abundances greater than 0.9 are considered pure. In principle, a larger buffer radius helps reduce estimation errors, assuming the spatial stationarity of endmember characteristics (composition, structure, and status) [24]. Our inspection of the resampled 10 m RGB imagery indicates that these endmember properties remain stable within a 50 m radius but may vary beyond this radius. Therefore, to balance estimation accuracy with spatial homogeneity, we conservatively selected a 9 × 9 square window. Based on the 10 m resolution of Sentinel-1A data, this implies a buffer radius of approximately 4.5 pixels (∼45 m), which falls safely within the observed stable zone. To estimate the non-negative endmember backscattering coefficients in Equation (14), we utilized the lsq_linear function from the SciPy library. The estimation results of endmember backscattering coefficients for different types of mixed pixels are shown in Figure 8. It should be noted that for the outermost five rows and five columns of the hyperspectral image in the test area, a 9 × 9 buffer could not be constructed. As a result, the endmember backscattering coefficients for the mixed pixels in these boundary regions were not estimated. Instead, their values were set to zero.

### 3.5. Endmember Types of the Test Area

**Figure 9:**
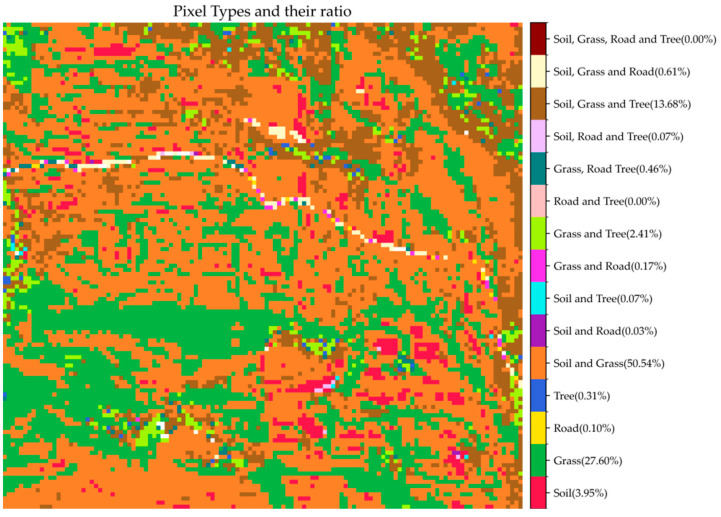
The proportion and spatial distribution of different types of pixels in the test area.

Prior to validation, it is essential to understand the types and spatial distribution of mixed pixels within the entire test area. To this end, we classified and encoded the mixed pixels. Based on the number of endmembers present in each pixel, the mixed pixels were categorized into four major groups: those containing 1, 2, 3, or 4 endmember types. Specifically, the number of possible combinations for each group is given by the binomial coefficients: $C_{4}^{1}$ for one endmember type, $C_{4}^{2}$ for two types, $C_{4}^{3}$ for three types, and $C_{4}^{4}$ for four types, resulting in a total of 15 distinct mixed pixel types. We summarized the number and proportion of each of these 15 types (Table 1) and visualized their spatial distribution across the study area (Figure 9).

As shown in Table 1, the mixed pixel types in the test area are primarily concentrated in Types 1, 4, 8, and 12. Among them, the most prevalent type is the mixture of soil and grass, which accounts for approximately 50% of the study area. Pure grass pixels constitute the second largest category, occupying approximately 27% of the area, while mixed pixels containing soil, grass, and trees cover more than 13% of the area. The remaining mixed pixel types are rare, each contributing less than 5% of the total area. According to the spatial distribution illustrated in Figure 9, mixed pixel types and distribution patterns are highly consistent with the land cover patterns shown in Figure 1. For example, pure grass pixels are mainly distributed in areas with higher grass coverage. In contrast, pixels containing both soil and grass are found in regions with lower grass density and partial soil exposure.

Considering that the validation process requires the buffer zone of the target mixed pixel to include pure pixels of all endmember types present in the mixed pixel itself, only Types 4 and 12—both involving at least two endmember classes and accounting for more than 5% of the study area—were eligible for evaluation. Therefore, Type 4 and 12 mixed pixels were selected to validate the accuracy of the estimated endmember backscatter coefficients.

### 3.6. Verification of the Estimated Endmember Backscattering Coefficients

Since the number of pure soil and grass pixels within the buffer zone of Type 4 mixed pixels is random and uncertain, not all Type 4 mixed pixels can be used to validate the estimated backscattering coefficients of grass and soil. Similarly, not all Type 12 mixed pixels can be used to validate estimates of grass, soil, and trees. Suppose the number of pure pixels corresponding to any endmember within the buffer falls below a certain threshold. In that case, random noise may render the backscatter coefficient of a pure pixel unreliable as a representative of the corresponding endmember’s actual backscatter response. Therefore, during the validation process, we first counted the number of pure soil and grass pixels within a 9 × 9 buffer around each Type 4 mixed pixel, and the number of pure soil, grass, and tree pixels within a 9 × 9 buffer around each Type 12 mixed pixel. To guarantee a sufficient sample size, an independent yet identical validation strategy was adopted for different endmembers. For instance, for a Type 4 mixed pixel, if its buffer contains more than six pure soil pixels, it is eligible for validating the accuracy of the estimated soil backscattering coefficient. Similarly, if the buffer contains more than six pure grass pixels, it can be used to validate the accuracy of the estimated grass backscattering coefficient.

**Figure 10:**
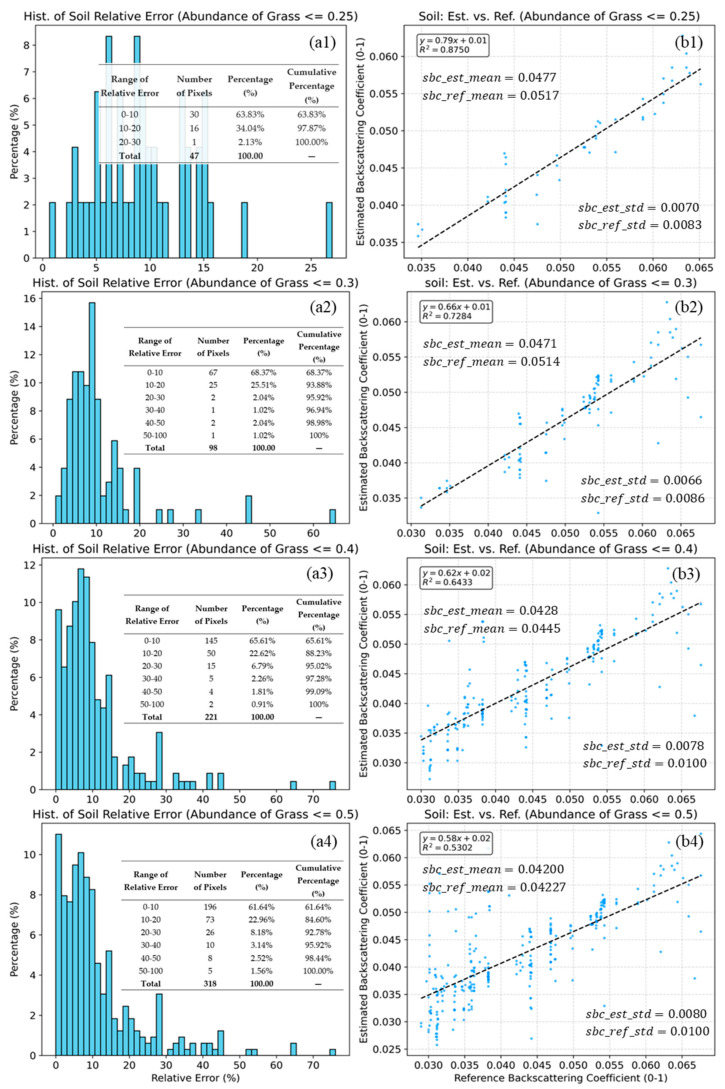
Histograms of relative error (**a1**–**a4**) and scatter plots of estimated vs. reference soil backscattering coefficients (**b1**–**b4**) for grass abundance thresholds of 0.25, 0.3, 0.4, and 0.5 (**b1**–**b4**). The dashed lines indicate the linear fits. Statistical indicators, including $R^{2}$, mean, standard deviation (**b1**–**b4**), and the relative error (**a1**–**a4**), are inset in the figure. The blue dots represent the sample points, while the grey dashed line indicates the linear fit between the predicted and reference values.

**Figure 11:**
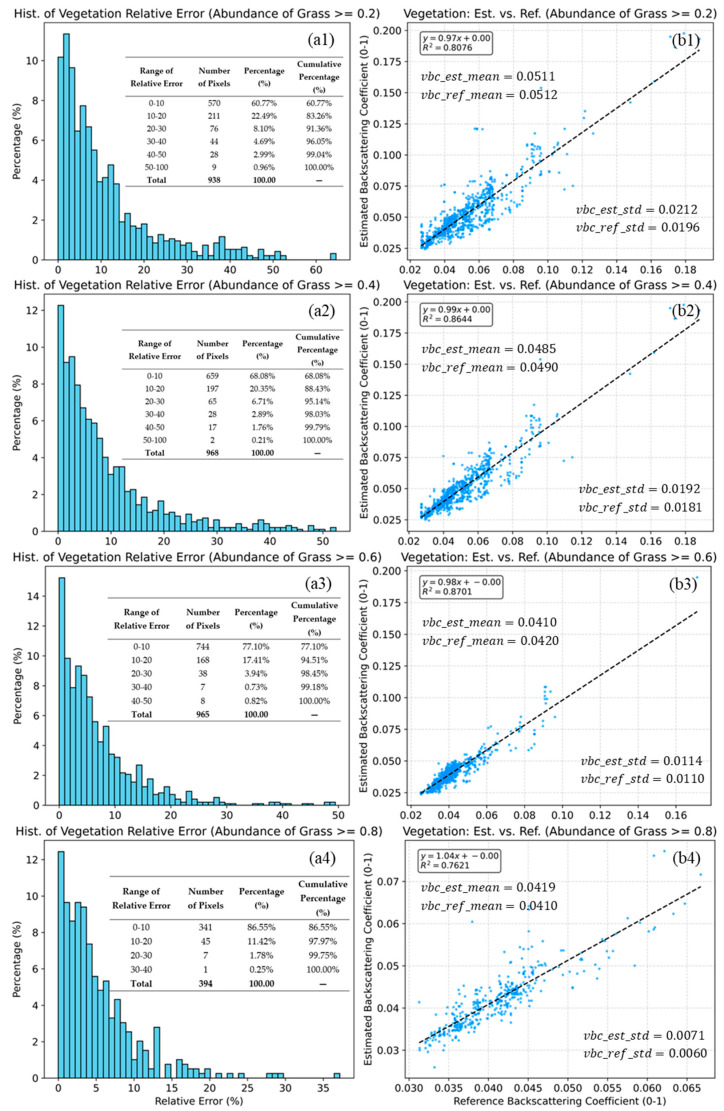
Histograms of relative error (**a1**–**a4**) and scatter plots of estimated vs. reference grass backscattering coefficients (**b1**–**b4**) for grass abundance thresholds of ≥0.2, 0.4, 0.6, and 0.8 (**b1**–**b4**). The dashed lines indicate the linear fits. Statistical indicators, including $R^{2}$, mean, standard deviation (**b1**–**b4**), and the relative error (**a1**–**a4**), are also inset in the figure. The blue dots (**b1**–**b4**) represent the sample points, while the grey dashed lines (**b1**–**b4**) indicate the linear fit be-tween the predicted and reference values.

During validation, we observed a notable phenomenon: the estimation accuracy of endmember backscattering coefficients varies significantly across different endmember abundances. Consequently, based on extensive experiments, we stratified the endmember abundance into specific ranges and validated the estimation accuracy within each range. For Type 4 mixed pixels, the validation results for the estimated soil and grass endmember backscattering coefficients are shown in Figure 10 and Figure 11, respectively. However, for Type 12 mixed pixels, the scarcity of pure soil and tree endmembers in the vicinity prevented the validation of the corresponding endmembers’ backscattering coefficients. Consequently, Type 12 mixed pixels were used solely to validate the grass endmember backscattering coefficients.

Figure 10 illustrates the validation results under four varying grass abundance thresholds (b1–b4). In the low-vegetation scenario (≤0.25), the method achieves the highest accuracy ($R^{2}\;$= 0.8750, Slope = 0.79). As the threshold relaxes to 0.50, the correlation decreases ($R^{2}\;$= 0.5302), and the slope declines to 0.58. The fact that the slope is consistently less than 1 indicates a compression of the dynamic range in the estimated results. Despite the reduced slope, the proposed method exhibits minimal systematic bias. As shown in Figure 10(b1–b4), the mean of the estimated soil backscattering coefficients (sbc_est_mean) aligns closely with the mean of reference soil backscattering coefficients (sbc_ref_mean) across all thresholds. Particularly at the ≤0.50 level, the difference is negligible (0.0420 vs. 0.0423), suggesting that the model remains globally statistically unbiased. In contrast to the means, a consistent disparity is observed in the variability. The standard deviation of the estimated soil backscattering coefficients (sbc_est_std) is consistently lower than that of the reference values (sbc_ref_std) (e.g., 0.0080 vs. 0.0100 at ≤0.50). This lower variability corroborates the “flattened slope” observed in the regression, confirming that the model tends to produce smoother estimates by filtering out extreme values or random noise present in the reference data. For Type 4 mixed pixels, the statistical results of the relative errors under different endmember abundances are shown in Figure 10(a1–a4).

Overall, the estimation accuracy remains robust across all tested thresholds. Even at the most relaxed threshold (≤0.50), the majority of pixels (61.64%) maintain a relative error within 10%, and 84.60% of pixels fall within the 20% margin. The precision is exceptionally high in sparse vegetation areas (≤0.25), where 97.87% of the pixels exhibit a relative error of less than 20%, with no errors exceeding 30%.

A comparison of Figure 10 and Figure 11 reveal that the number of sample points in Figure 10 is significantly smaller than that in Figure 11. This disparity is attributed to the characteristics of the grassland. In such environments, pure grass pixels are naturally far more abundant than pure bare soil pixels.

Figure 11 presents the validation results for the grass endmember under four increasing abundance thresholds (≥0.20, 0.40, 0.60, and 0.80). In contrast to soil backscattering coefficient estimation, the model’s performance for vegetation backscattering coefficient retrieval improves or remains highly stable as the vegetation coverage increases.

The method demonstrates strong agreement across all intervals. Even at the lowest threshold (≥0.20), the model achieves a high correlation ($R^{2}\;$= 0.8076). This correlation peaks at ≥0.60, with an $R^{2}$ of 0.8701. A remarkable feature is that the regression slopes are consistently close to 1.0, ranging from 0.97 to 1.04, indicating that the model correctly captures the magnitude of vegetation backscattering coefficient from sparse to dense cover conditions.

The statistical results shown in Figure 11(b1–b4) further confirm the robustness of the retrieval. The method exhibits negligible systematic bias. The mean of the estimated values (vbc_est_mean) is almost identical to the reference mean (vbc_ref_mean) in all cases (e.g., 0.0511 vs. 0.0512 at ≥0.20). The standard deviations of the estimated and reference values are highly comparable (e.g., 0.0212 vs. 0.0196 at ≥0.20). The slightly high difference in standard deviations suggests that the model preserves the natural variability of the vegetation signal without over-smoothing.

The estimation accuracy is positively correlated with grass abundance. At the lowest threshold (≥0.20), 83.26% of pixels already fall within the 20% relative error margin. As the abundance constraint tightens to ≥0.60 and ≥0.80, the accuracy becomes exceptional. At ≥0.60, 94.51% of pixels have errors under 20%. At the highest abundance level (≥0.80), this figure rises to 97.97%, with significant errors (>30%) virtually eliminated (cumulative 0.25%).

**Figure 12:**
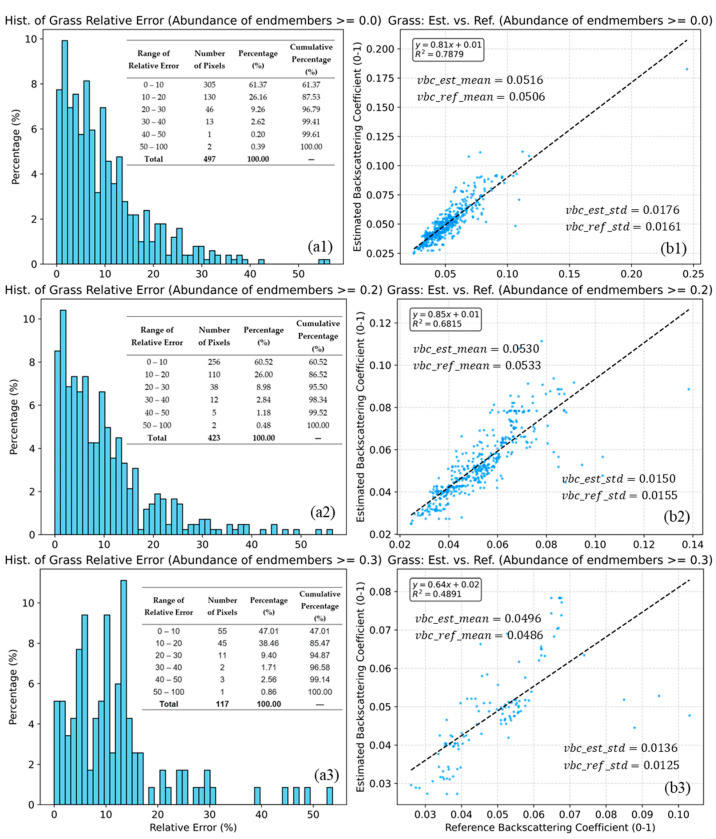
Histograms of relative error (**a1**–**a3**) and scatter plots of estimated vs. reference grass backscattering coefficients (**b1**–**b3**) for grass abundance thresholds of 0.25, 0.3, 0.4, and 0.5 (**b1**–**b3**). The dashed lines indicate the linear fits. Statistical indicators, including $R^{2}$, mean, standard deviation, and the relative error (**a1**–**a3**), are also inset in the figure. The blue dots (**b1**–**b3**) represent the sample points, while the grey dashed lines (**b1**–**b3**) indicate the linear fit be-tween the predicted and reference values.

Figure 12 presents the validation results for the grass endmember backscattering coefficients in Type 12 mixed pixels. The model achieves its best performance when no restrictions are imposed on the endmember abundance ranges ($R^{2}=0.7879$, with approximately 88% of pixels having relative errors within the 0–20% range). However, the model’s accuracy declines as endmember abundances become more similar. For instance, when all endmember abundances exceed 0.2, the $R^{2}$ decreases to 0.6815, and the proportion of pixels with relative errors in the 0–20% range falls to approximately 87%. Furthermore, when all endmember abundances are very close, the $R^{2}$ drops to 0.4891, and the proportion of pixels within the 0–20% relative error range decreases to about 85%. Nevertheless, the differences between the mean values of the estimated and reference grass backscattering coefficients are negligible (maximum difference of 0.001), as are the differences between their standard deviations (maximum difference of 0.0015).

## 4. Discussion

### 4.1. Accuracy of Endmember Abundance Estimation

The accuracy of endmember abundance estimation was evaluated from both qualitative and quantitative perspectives. Overall, the spatial distributions of soil, grass, road, and tree abundances in the abundance maps (Figure 6) show a high degree of consistency with the distributions of these land cover types in Figure 1. Apart from artificial objects, the environmental parameters of natural soils that influence vegetation growth typically exhibit gentle and continuous spatial variations. Consequently, the biomass supported by the soil also varies gently and continuously across space, implying that vegetation cover should not experience drastic spatial changes; instead, it is expected to vary similarly and continuously. For each endmember (soil, grass, or tree) abundance map, the spatial continuity of the abundance values corresponds well to the expected continuity of the associated endmember cover fraction. In particular, the bright areas in the abundance maps (Figure 6) closely match regions with higher coverage of the corresponding land cover types in Figure 1.

To quantitatively evaluate the accuracy of endmember abundance estimation in this study, we conducted an extensive review of the literature related to hyperspectral unmixing. The results of this review indicate that, when applied to the same hyperspectral dataset, deep learning-based unmixing methods generally outperform the FCLS method used in this study. However, the accuracy of the FCLS method primarily depends on the selection of pure endmember spectral signatures. By adopting a novel approach that combines visual interpretation with NDVI-based density segmentation, the RMSE of hyperspectral unmixing achieved in this study, while slightly lower than that of deep learning methods, is nonetheless very close to that of deep learning methods, which demonstrates that the endmember abundance results derived from hyperspectral unmixing in this study are reliable (Figure 7) [52,53,54,55].

### 4.2. Performance Evaluation of Endmember Backscattering Coefficient Estimation Schemes

#### 4.2.1. Qualitative Evaluation of Endmember Backscattering Coefficient Estimation Result

As shown in Figure 8a, when soil abundance is lower than 5%, its backscattering coefficient is set to 0. The soil backscattering coefficients across most grass-covered areas are relatively low, whereas a significant increase is observed in areas adjacent to trees, likely because soils near trees have higher moisture and inorganic salt content than soils in grassland areas, leading to a notable increase in their backscattering coefficients. Trees generally require more water and nutrients for growth than grasses, which supports the observed spatial distribution of the soil backscattering coefficients. Moreover, the estimated soil backscattering coefficients exhibit good spatial continuity, meaning their variations across space are relatively gentle, consistent with the spatial distribution patterns of soil properties, such as type and condition, that govern backscattering behavior. These results demonstrate that the estimated soil backscattering coefficients possess good spatial consistency and are physically reasonable.

Due to mixed pixel types that do not contain grass endmembers (types 0, 2, 3, 5, 6, and 11), there are invalid-value regions in the grass backscattering coefficient map, as shown in Figure 8b. By carefully checking the spatial distribution of grass endmember backscattering coefficients in Figure 8b, it can be observed that most areas containing grass endmembers exhibit relatively low backscattering coefficients. In regions where tree endmembers are also present (as shown in Figure 8b,d), the grass backscattering coefficients show a moderate increase, although not as pronounced as the increase observed for soil backscattering coefficients. This phenomenon may be attributed to soils near trees having higher moisture and inorganic salt content than grassland soils, which promote more vigorous grass growth. Consequently, the grass’s biomass and water content in these areas are slightly higher than in regions without tree endmembers, leading to a moderate increase in the grass backscattering coefficients. Like the spatial distribution of soil backscattering coefficients, the estimated grass backscattering coefficients exhibit good spatial continuity, consistent with the spatial patterns of factors such as grass type, structure, and growth status that influence backscattering behavior. These results indicate that the estimated grass backscattering coefficients also possess good spatial consistency and are physically meaningful.

Since mixed pixels containing roads are usually found at the edges of roads, a large number of invalid-value regions appear in the road’s backscattering coefficient map. However, as shown in Figure 8c, some mixed pixels containing road endmembers remain along roads, which may be due to concrete surfaces in the area or soils with spectral characteristics similar to those of the roads. As expected, the backscattering coefficients across the road area are relatively low because the road surface is electromagnetically smooth to C-band waves. Most of the energy is reflected specularly, resulting in minimal backscattered energy. The low backscattering coefficients are consistent with the road’s geometric properties.

The tree end member is absent in most of the test area, leading to many invalid values in the tree backscattering coefficient map. It can be observed that the backscattering coefficients of tree endmembers across the entire study area are generally low, though slightly higher than those of the corresponding grass regions, which is because the biomass and water content of tree endmembers are typically higher than those of grass endmembers, resulting in higher backscattering energy for tree endmembers. Furthermore, the backscattering coefficients are significantly higher in regions with higher tree endmember abundance than those with lower tree endmember abundance, which could be attributed to the denser tree coverage. As trees become dense, tree trunks, branches, and leaves increase, leading to stronger backscattering energy. Consequently, areas with higher tree endmember abundance exhibit relatively higher backscattering energy. Lastly, the continuous spatial distribution of tree endmember backscattering coefficients further indicates that the estimated results have good spatial consistency.

#### 4.2.2. Quantitative Evaluation of Endmember Backscattering Coefficient Estimation Result

The scatter plots in Figure 10 and Figure 11 demonstrate that the proposed model generally achieves relatively high accuracy in estimating endmember backscattering coefficients. However, its performance shows significant variation across different vegetation cover levels and endmember types.

The declining trend in $R^{2}$, combined with the relative error statistics in Figure 10(b1–b4), indicates that the model’s ability to retrieve soil backscattering coefficients accurately degrades significantly as the proportion of pixels with high vegetation coverage increases in the validation dataset. The model exhibits optimal performance when vegetation coverage is below 0.25, characterized by a high $R^{2}$ ($R^{2}\approx 0.88$) and low relative error. Conversely, scenarios with extremely low vegetation coverage (e.g., <20%) were excluded from this evaluation due to the scarcity of sample points, which would render statistical significance impossible.

The $R^{2}$ value, combined with the relative error statistics in Figure 11(b1) (where vegetation coverage ≥ 0.2), indicates that for natural grasslands (with coverage typically between 0.2 and 0.8), the model is capable of accurately retrieving the grass endmember backscattering coefficient while effectively capturing its spatial variation trends. Furthermore, comparisons across Figure 11(b1–b4) suggest that the model’s performance may fluctuate—either improving or declining—when the vegetation coverage is concentrated within specific intervals. Specifically, as shown in Figure 11, when vegetation coverage is concentrated in the 40–80% range (noting that pure pixels exceeding 80% are relatively scarce), the model achieves an $R^{2}$ of 0.86. This value peaks at 0.87 for the 60–80% range but drops to 0.76 when coverage exceeds 80%. These results demonstrate that the model’s ability to retrieve grass backscattering coefficients varies with vegetation cover.

Regarding the estimation of soil backscattering coefficients, the superior retrieval accuracy observed under low vegetation coverage can be attributed to the two-way attenuation of electromagnetic signals by the vegetation canopy.

In the validation process, pure soil pixels within the buffer of mixed pixels were utilized as the reference. Being devoid of grass cover, these pure pixels are free from canopy attenuation for both incident and scattered waves. Consequently, after atmospheric correction, their backscattering coefficients are fundamentally determined by the soil’s physicochemical parameters. Assuming negligible differences in physicochemical properties between the soil in pure pixels and that within mixed pixels (uncertainties arising from actual discrepancies are discussed in Section 4.3), the average backscattering power of the soil component in both cases should ideally be identical.

However, for the soil component within mixed pixels, the current model does not compensate for the power loss due to the attenuation of the incident wave penetrating the grass layer, nor for the attenuation of the backscattered wave exiting the canopy. As a result, the calculation overestimates the incident power relative to the power actually reaching the soil surface. In contrast, the received backscattered power is underestimated relative to the true soil backscatter, leading to an underestimation of the retrieved soil backscattering coefficient. Consequently, as vegetation coverage decreases, this two-way attenuation effect weakens, enabling more accurate retrieval, conversely, as vegetation coverage increases, attenuation intensifies, causing the estimated backscattering coefficients to deviate more from the actual soil backscattering coefficients.

For the estimation of vegetation backscattering coefficients, the model performs consistently well across the 0.2–0.8 coverage range. The model’s good performance is primarily due to neither the incident nor the backscattered power from the vegetation component being attenuated. The observed performance variations across different abundance ranges can be attributed to multiple factors. The significant rise in $R^{2}$ from Figure 11(b1, b2) (0.81 to 0.86) is likely due to the low SNR of low grass coverage pixels. In low-vegetation-abundance pixels, the majority of the echo energy originates from the soil. Retrieving the grass backscattering power essentially involves extracting a weak signal from a dominant background noise (soil scattering and speckle). According to the law of error propagation, minor errors in soil abundance estimation or weak measurement noise are propagated to the estimated grass coefficient, leading to random fluctuations around the actual value. This aligns with the dispersed scatter pattern observed in Figure 11(b1). When vegetation coverage is high and concentrated within a narrow range (e.g., ≥0.8 in Figure 11(b4)), the reference grass backscattering coefficients are confined to a limited interval (0.03–0.065). The standard deviation decreases to 0.0060—a reduction of 0.0050 compared to the ≥0.6 scenario, nearly halving the variability. While the relative error decreases only marginally (Figure 11(b4)), this narrower data range inevitably leads to a lower $R^{2}$.

Figure 12 demonstrates the model’s capability to estimate backscattering coefficients in mixed pixels containing multiple endmembers. As illustrated in Figure 10, the lower relative errors and higher $R^{2}$ indicate that the model remains stable as the number of endmembers increases, maintaining high estimation accuracy. However, it should be noted that when endmember abundances are similar (as shown in Figure 10(b3)), the model’s accuracy in estimating endmember backscattering coefficients may experience some decline.

It is imperative to note that the model may become inapplicable in certain specific scenarios, such as when different endmembers within the buffer zone exhibit nearly identical backscattering coefficients. In such cases, the composite backscatter vector b (Equation (11)) is no longer dominated by the differences in endmember abundances but is instead driven by observational noise in the backscattering coefficients. Consequently, the solutions for the endmember backscattering coefficients become unreliable. Such scenarios can indeed occur in natural environments; examples include areas where dense vegetation grows on soil with extremely high water content, or boundary regions between concrete roads and water bodies.

In summary, the model’s retrieval performance exhibits distinct characteristics depending on the endmember and coverage conditions. First, for soil retrieval, the model performs optimally in low-vegetation areas (<0.25) and accurately captures spatial trends in soil physicochemical parameters. However, as coverage increases, vegetation’s increased attenuation progressively degrades both estimation accuracy and the ability to characterize soil spatiotemporal changes. Second, for vegetation retrieval, the model demonstrates high precision across the 0.2–0.8 coverage range, effectively reflecting growth status. It is worth noting that while the performance is generally robust, minor fluctuations may occur across different intervals, and a slight decline in performance is observed when coverage is concentrated within a narrow range. Moreover, a slight degradation in the model’s performance for estimating endmember backscattering coefficients is observed as the number of endmembers increases.

### 4.3. Uncertainty in Model Assessment Arising from the Validation Scheme

To explicitly illustrate the potential model-evaluation bias induced by this validation strategy, we consider a mixed-pixel scenario containing soil and grass to demonstrate the uncertainty. For example, the target point P is surrounded by mixed pixels, pure grass pixels, and pure soil pixels.

The validity of using the average backscattering power of pure grass pixels as a proxy for the grass component within mixed pixels hinges on whether the composition, structure, and growth status (determinants of the backscattering coefficient) of the grass in pure pixels are substantially identical to those in the mixed pixels. In reality, high grass abundance implies high plant density, intensifying competition for nutrients and light, potentially resulting in reduced individual plant biomass. Conversely, under lower vegetation coverage, the reduced plant density alleviates this competition, leading to higher individual biomass. Driven by these countervailing mechanisms, the estimated backscattering coefficient of the grass endmember within mixed pixels may deviate slightly—either higher or lower—from that of pure grass endmembers. This interpretation is consistent with the observations (the slope varies between 0.97 and 1.04, closely approximating unity) presented in Figure 11(b1–b4).

The volumetric water content in pure soil pixels is generally lower than in mixed pixels; furthermore, soil nutrients (e.g., inorganic salts) that facilitate grass growth are likely lower in pure barren soil than in soil within mixed vegetation pixels. Therefore, the backscattering coefficient of pure soil pixels should be lower than that of the soil component in mixed pixels. However, the slope range (0.58–0.79) observed in Figure 10(b1–b4) clearly indicates that the estimated soil backscattering coefficients are generally lower than the actual reference values. This systematic underestimation is primarily due to the dominant two-way attenuation effect of vegetation among the aforementioned factors influencing soil backscattering. Nevertheless, our previous work indicated that under high vegetation cover—where soil moisture is typically high, and soil backscattering exceeds vegetation backscattering—the estimated soil backscattering coefficient was significantly higher than that of the mixed pixel [24]. This observation, to a certain extent, effectively validates the reliability of the proposed scheme.

### 4.4. Application Prospects and Future Work of This Study

According to the classification standards of the International Geosphere-Biosphere Programme (IGBP) and the Food and Agriculture Organization (FAO), MODIS vegetation fractional cover data indicate distinct threshold characteristics across different biomes. Specifically, tropical rainforests typically exhibit coverage exceeding 90% (often approaching 100%); temperate and boreal forests range from 60% to 90%; savannas generally fall between 30% and 60%; shrublands range from 30% to 70%; natural grasslands vary between 20% and 80%; desert regions typically show coverage below 5%; and tundra coverage ranges from 40% to 80%. Drawing upon the conclusions regarding endmember backscattering coefficient retrieval in Section 4.2, the proposed model holds significant potential for retrieving vegetation backscattering coefficients in temperate/boreal forests, savannas, shrublands, natural grasslands, and tundra. This capability would enable the monitoring of spatiotemporal trends in the physicochemical parameters of these vegetation types. Furthermore, in desert regions, the model can characterize the desertification process by accurately retrieving soil backscattering coefficients—a hypothesis validated in our previous work (noting that in desertification environments, soil volumetric water content is typically below 5%, rendering backscattering primarily dependent on surface roughness) [24]. In another study, based on the soil and vegetation backscattering coefficients estimated by this model, we successfully decomposed and estimated the radar temporal decorrelation signals of mixed pixels (containing only soil and vegetation). Consequently, we were able to characterize the wind erosion intensity across the desiccated bed of the Aral Sea [56]. In future research, we aim to mitigate the two-way attenuation effect of vegetation on soil backscattering retrieval under moderate-to-high vegetation coverage, thereby improving the accuracy of soil backscattering coefficient estimates. Additionally, we intend to conduct rigorous testing across diverse environmental conditions to evaluate the model’s applicability and generalizability comprehensively.

The current scarcity of hyperspectral data represents the most critical constraint on the application of the proposed method; however, multispectral data can also be attempted for endmember abundance estimation. If the accuracy of endmember abundances derived from multispectral data is sufficiently high, the limitations imposed by the shortage of hyperspectral data would be significantly mitigated. Furthermore, in regions consisting solely of soil and a single vegetation type, fractional vegetation cover (FVC) methods remain a viable option for estimating endmember abundances, thereby facilitating the subsequent estimation of soil and vegetation backscattering coefficients. It is anticipated that in the near future, as hyperspectral data become increasingly available, constraints on estimating endmember backscattering coefficients will be further alleviated.

## 5. Conclusions

In this study, a novel scheme for estimating endmember backscattering coefficients within mixed pixels was proposed by integrating the MBCD model with spectral mixture analysis. Based on the validation of the estimation results and the comprehensive evaluation of the model, the following conclusions are drawn:

(1) The newly developed MBCD model can effectively decompose the backscattering contributions of endmembers within mixed pixels (containing multiple endmember types).
(2) Under low vegetation coverage conditions (below 25%), the proposed scheme yields accurate estimates for soil endmember backscattering coefficients, achieving an $R^{2}$ of 0.88, with 98% of samples showing a relative error within 20%. However, as vegetation coverage increases, the two-way attenuation effect of vegetation on soil backscattering energy becomes more pronounced, leading to a slight decline in the scheme’s performance.
(3) The model is capable of relatively accurate estimation of grass endmember backscattering coefficients in grassland (with vegetation coverage between 20% and 80%), achieving an $R^{2}$ of 0.81, with 83% of samples having a relative error within 20%. Nevertheless, the model exhibits sensitivity to vegetation coverage, showing minor fluctuations (improvements or declines) in performance across different coverage ranges.
(4) The model remains robust in estimating backscattering coefficients even as the number of endmember types within a mixed pixel increases.
(5) The model may become ineffective when the backscattering coefficients of the endmembers within a mixed pixel are highly similar, resulting in unreliable estimation results.

## Figures and Tables

**Table 1:** Types, coding, and proportions of pixels in the test area.

Endmembers Numbers in Pixel	Pixel Types	Endmember Coding	Pixels Number	Ratio (%)
1	Soil	0	577	3.95
	Grass	1	4037	27.60
	Road	2	14	0.10
	Tree	3	46	0.31
2	Soil and Grass	4	7392	50.54
	Soil and Road	5	4	0.03
	Soil and Tree	6	10	0.07
	Grass and Road	7	25	0.17
	Grass and Tree	8	353	2.41
	Road and Tree	9	0	0.00
3	Grass, Road Tree	10	68	0.46
	Soil, Road and Tree	11	10	0.07
	Soil, Grass and Tree	12	2000	13.68
	Soil, Grass and Road	13	89	0.61
4	Soil, Grass, Road and Tree	14	0	0

## Data Availability

The data presented in this study are available on request from the corresponding author due to privacy.

## Abbreviations

The following abbreviations are used in this manuscript:

SAR	synthetic aperture radar
DVP	Depolarized Volume Power
AVP	Anisotropic Volume Power
LAI	Leaf Area Index
PPI	Pixel Purity Index
NEON	National Ecological Observatory Network
AOP	Airborne Observation Platform
VSWIR	visible to shortwave infrared
QA	quality assurance
BRDF	Bidirectional Reflectance Distribution Function
GEE	Google Earth Engine
GRD	Ground Range Detected
SRTM	Shuttle Radar Topography Mission
DEM	Digital Elevation Model
dB	decibel
SVD	singular value decomposition
NDVI	Normalized Difference Vegetation Index
FCLS	Fully Constrained Least Squares
RMSE	root mean square error
